# From Knockouts to Networks: Establishing Direct Cause-Effect Relationships through Graph Analysis

**DOI:** 10.1371/journal.pone.0012912

**Published:** 2010-10-11

**Authors:** Andrea Pinna, Nicola Soranzo, Alberto de la Fuente

**Affiliations:** Center for Advanced Studies, Research and Development (CRS4) Bioinformatica, Pula, Italy; Center for Genomic Regulation, Spain

## Abstract

**Background:**

Reverse-engineering gene networks from expression profiles is a difficult problem for which a multitude of techniques have been developed over the last decade. The yearly organized DREAM challenges allow for a fair evaluation and unbiased comparison of these methods.

**Results:**

We propose an inference algorithm that combines confidence matrices, computed as the standard scores from single-gene knockout data, with the down-ranking of feed-forward edges. Substantial improvements on the predictions can be obtained after the execution of this second step.

**Conclusions:**

Our algorithm was awarded the best overall performance at the DREAM4 In Silico 100-gene network sub-challenge, proving to be effective in inferring medium-size gene regulatory networks. This success demonstrates once again the decisive importance of gene expression data obtained after systematic gene perturbations and highlights the usefulness of graph analysis to increase the reliability of inference.

## Introduction

Reverse engineering is an interesting area of research currently receiving a lot of attentions from the Systems Biology community. In fact, reconstructed biomolecular networks may allow researchers to understand the molecular basis of complex traits and diseases [1], as well as the discovery of direct drug targets [2].

Gene expression data have been prevailing over protein and metabolite activity data, because of the relative ease and unified way to measure RNA levels, and this disproportion will be further increased due to the appearance of gene expression measurements techniques based on novel sequencing technologies, e.g. [3]. Therefore, the concept of gene network (GN) is of high importance for the purpose of describing the regulatory networks inside living cells. GNs are abstract models of gene communication with nodes representing the gene activities (gene expression levels, mRNA concentrations), and directed edges representing causal influences [4], [5]. The causal influence of gene A on gene B could be due to the transcription activation of gene B by the protein product of gene A upon binding to gene B's promoter sequence (as in a transcription factor–target relationship), but also be due to more complicated processes, such as gene A encoding a metabolic enzyme producing a metabolite which in turn regulates the transcription of gene B. These detailed biochemical events are hidden to the observed set of variables (gene expression levels) and their effects will merely result in an observable causal effect A
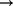
B. Undirected edges in GNs are present due to unmeasured confounding variables. GNs are context specific: the regulatory structure among genes depends on the developmental stage, cell type, environment, genotype and disease state. For a comprehensive discussion on the nature of GNs please refer to [5].

As a precise definition of GNs is missing in current literature we here provide (one possible) formal definition.

### Definition

A *gene network* is a mixed graph 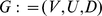 over a set 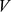 of nodes, corresponding to gene activities, with unordered pairs 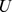, the undirected edges, and ordered pairs 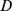, the directed edges. A directed edge 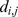 from 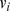 to 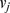 is present if and only if a causal effect runs from node 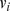 to 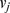 and there exist no nodes or subsets of nodes in 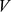 that are intermediating the causal influence (it may be mediated by hidden variables, i.e. variables not in 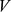). An undirected edge 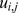 between nodes 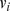 and 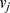 is present if and only if gene activities 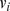 and 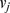 are associated by other means than a direct causal influence, and there exist no nodes or subsets of nodes in 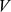 that explain that association (i.e. it is caused by a variable hidden to 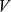).

Depending on the available measurements, different inference techniques can be employed. In case of experiments without targeted perturbations (“observational studies”, such as gene expression data collected over a group of similar individuals, typically done in the context of a disease) the expression profiles can be analyzed to build a undirected graph whose nodes are the genes, and whose edges represent the presence of significant associations. Without targeted perturbations it is not generally possible to infer directions of the edges. A wide variety of techniques for constructing such undirected co-expression networks has been proposed, typically based on marginal associations [6], conditional associations [7]–[9] or information theory [10]. Under some assumptions it is theoretically possible to decide the orientation of the edges using this type of data [11], [12], but unfortunately these assumptions (such as acyclicity of the network and absence of confounding factors) are very unlikely to be met in the present context.

On the other hand, targeted perturbations (e.g. systematic single-gene gene knockouts, overexpressions) are needed to enable causal inference, and the reconstruction of the directed structure of GNs. Many techniques for constructing GNs have been proposed of which the most popular techniques are based on ordinary differential equations [13]–[15] or Bayesian networks [16].

The performance of these techniques needs to be evaluated and compared [17], [18], and this can be accomplished by applying different inference methods to the data obtained from biomolecular networks of which the structure is assumed to be known *a priori*, i.e. “gold standard networks” [19]–[21]. However, real world biomolecular networks are mostly unknown. Even the most studied biomolecular networks are not only plagued by false positives, but suffer even worse from false negatives: they are largely incomplete [22]. Consequently such networks cannot be deemed as totally reliable benchmarks to compare inference algorithms. Therefore, it has been suggested to use data simulated with dynamical models of GNs, i.e. *in silico* data. In this case the underlying networks are precisely known and thus allow for thorough evaluation and comparison of reverse-engineering algorithms [23], [24]. Obviously, the relevance of evaluations on *in silico* data strongly depends on the realism of the simulation system, e.g. the network topology, the type of mathematical model, the type of kinetic functions, the noise model, etc.

The outline of the paper is the following: we first describe the DREAM4 *In Silico* Network challenges, then explain the inference algorithm we devised and applied to the DREAM 4 data, followed by a description of the GN simulator we developed to generate additional synthetic networks and data. Then, we show the evaluations of variants of our algorithm on both the DREAM3 *In Silico* benchmarks and the additional simulated datasets. We also present the results of re-analysis of the DREAM4 *In Silico* benchmarks, which we were able to perform after the gold standard networks were released. We conclude with a discussion of the method, data and future steps to be made.

### DREAM4 *In Silico* Network challenge

The Dialogue for Reverse Engineering Assessments and Methods (DREAM) is an international initiative with the aim of evaluating methods for biomolecular network inference in an unbiased way [17], [18]. Evaluations proceed through organized competitions on a yearly basis in which teams from all over the world participate. For the 4th edition of DREAM in 2009, the organizers proposed three different challenges. Our team participated in the second one, the *In Silico* Network challenge, which asked to infer directed GNs from simulated data. The challenge was, in turn, divided into three sub-challenges, respectively named *InSilico_Size10*, *InSilico_Size100*, and *InSilico_Size100_Multifactorial*.

These sub-challenges differed, as their names suggest, in the network size and the type of data provided. In the first sub-challenge the partecipants had to predict the topology of five 10-gene networks, and were provided with steady state gene expression levels from wild-type, knockouts, knockdowns, multifactorial perturbations, and time series data. The second sub-challenge concerned instead five 100-gene networks, with the same type of available data except the multifactorial perturbations. The third sub-challenge involved five other 100-gene networks provided with multifactorial perturbations data only. The contestants were challenged to predict the network structures underlying the above data, i.e. assigning a level of confidence for the presence of each possible edge.

We here provide a brief description of the available data provided to the DREAM 4 partecipants. The number of genes in the network is denoted by 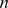. The *wild-type* file contained the 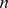 steady-state levels of the unperturbed network. The *knockout* data (see an example in Table 1) consisted of 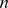 rows with 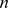 steady-state values, each obtained after deleting one of the 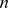 genes. The *knockdown* data were similar to the above, but were obtained by halving the transcription rate constant of one gene at a time instead of setting it to zero. The *multifactorial perturbations* data consisted of steady-state levels of small fluctuations of the values of all transcription rate constants simultaneously. The *time series* file contained trajectories of gene activity levels starting from the wild-type steady state to a perturbed state, and from the perturbed state back to the wild-type state upon removing the perturbations.

**Table 1:** Sample of knockout data.

	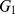	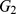	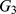	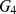	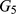
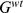	0.14	0.89	0.01	0.87	0.14
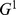	0.00	0.96	0.00	0.86	0.06
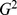	0.68	0.00	0.04	0.90	0.05
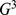	0.17	0.86	0.00	0.88	0.02
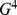	0.13	0.86	0.08	0.00	0.09
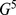	0.12	0.78	0.09	0.91	0.00

This is an example of the provided knockout data, related to an example 5-gene network. The first row contains the ‘wild-type’ (unperturbed) gene activities, while the others contain the gene activities due to the knockout of the gene indicated on the left. A knocked-out gene has null expression. Data are affected by noise, but certain relationships are apparent: 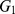 is likely to be regulated by (or at least downstream of) 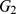, since the steady state value of 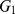 responds strongly to perturbing 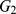: 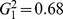 noticeably differs from 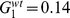.

The network topologies to be inferred were generated by the organizers by extracting 10- or 100-node subnetworks from transcriptional regulatory networks of *E. coli* and *S. cerevisiae*, with preferential selection of parts containing cycles (but no self-interactions).

The challenge description mentioned also that the data was simulated through a dynamical model describing both independent and synergistic gene regulation, which included both gene and protein expression (but only the gene expression data was provided to the partecipants). Internal noise was modeled through stochastic (Langevin) differential equations, and measurement noise was added to the simulated gene expression levels. Networks and data were generated by the *GeneNetWeaver 2.0* software [25], which was published only after the DREAM4 conclusion.

## Methods

### Algorithm

The aim of these challenges was the prediction of the (directed and unsigned) network structures. How can we infer such gene regulatory networks? While the time-series data could be used for this purpose, the lack of protein measurements would make it difficult to infer relationships between gene activities from time dynamics: the protein dynamics causes delays between the gene expression dynamics. Therefore, we resorted to the steady state levels, in particular to the knockout datasets, where the perturbations and the relative responses were stronger.

From this kind of data it is very easy to infer a so-called causal influence network: genes whose steady state values change as a result of a single-gene knockout are likely to be downstream of the perturbed gene [26], [27]. Most causal relationships (both activating and inhibiting) due to the knocked-out gene could be immediately recognized from the data table (e.g. Table 1), unless the influence is particularly weak and then overwhelmed by noise, or its effect is mitigated by other connections. This approach will not infer spurious relationships between co-regulated genes, which is instead a well-known problem of algorithms based on expression similarity (e.g. correlation) [7].

However, some of the edges of a causal influence network may be indirect, i.e. mediated by other (measured) gene activities [26]. The remaining task is thus to distinguish direct from indirect relationships. To accomplish this, we developed an algorithm consisting of two main steps: through statistical measures, a first estimate of the confidence of each possible edge is obtained directly from the available knockout data; then, by down-ranking the feed-forward edges, a refined prediction is given.

In the first step we quantify the importance of the responses of the gene activities toward single-gene perturbations and so how likely it is for each gene to be downstream of the perturbed genes. Let 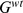 be the vector of wild-type gene expression, and let 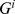 be the vector of gene activity steady-states obtained by knocking out gene 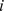. To obtain the initial predictions, we evaluated four possible different confidence matrices W in which elements 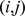 reflects the confidence in the existence of the edge 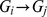:

#### Deviation matrix, 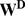


The confidence of edge 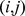 is simply estimated by the absolute value of the deviation from wild type of the expression of gene 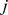 after the knockout of gene 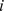: 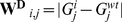. The larger the deviation the higher the confidence we have that 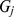 is downstream of the perturbed 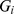.

#### Normalized deviation matrix, 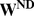


As the absolute values of the steady state gene activities vary drastically (e.g. 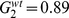 and 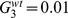 in Table 1) it might be more appropriate to consider the relative deviations. Each column of the deviation matrix is normalized by the corresponding wild type: 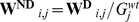.

#### Z-score on deviation matrix, 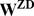


A more statistically motivated measure is the z-score, which indicates how many standard deviations 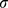 an observation is far from the mean 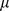 of a whole set of measurements. In this case, for each gene 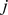 we calculate 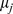 and 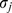 using the deviations from wild type after each knockout (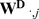):
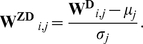



#### Z-score on raw data matrix, 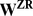


**Figure 1:**
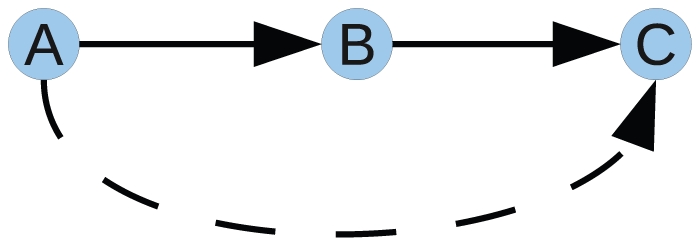
Feed-forward loop. The edge between gene A and gene C might be erroneously predicted as the causal effect of gene A on gene C could in principle be explained by the indirect path through gene B.

As both 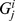 and 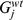 are noisy values, it may be better to consider raw expression values rather than deviations from the steady state values (subtracting a noisy value from another noisy value results in a even noisier value). Therefore, for each gene 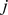 we calculate 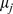 and 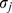 using the steady-state values after each knockout (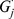):
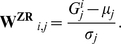



Once a first prediction of the network has been calculated with one of the above methods, the second step of the inference algorithm comes into play. The logic behind this second step is also plain and simple. First, based on a threshold value on the derived confidence matrix, a network is obtained. This network contains edges which represent causal influences between the genes, which may be direct or indirect. The “true” network is thus embedded in this initial causal influence network and could be derived by removing edges (edges can not be added as they create causal influences not supported by the perturbation experiments). We recognize that certain edges can be removed without removing the causal influences: the edge from gene A to gene C could be removed if there is at least one additional path from gene A to C in the network [26]. The additional path(s) could explain the causal effect of gene A on C and therefore we have reduced confidence in the existence of the direct edge from A to C. Figure 1 provides an example of a feed-forward loop from which an edge could be removed.

Our down-ranking algorithm systematically checks for paths through the initial networks and recognizes which edges can be removed (potentially indirect) and which edges can not be removed (these must be direct as removing them would result in a network missing one or more of the observed causal influences). Note that cyclic components in the networks are fully connected, as each gene in a cycle has a causal influence on all other genes in the cycle. Determining which edges in a cyclic component can be removed without removing causal paths depends on the order in which the edges are removed. Therefore, we decided not to touch any of the edges in cyclic components. We emphasize that we do not believe that the sparsest network possible is most biologically realistic. In fact, it is widely recognized that biomolecular networks are enriched in feed-forward loops [28]. However the absolute frequencies of their occurrence in the networks is much lower than that of the linear path motif (A
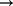
B
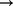
C). Therefore, it is reasonable to assume that down-ranking these edges improves the reliability of the network inference.

**Figure 2:**
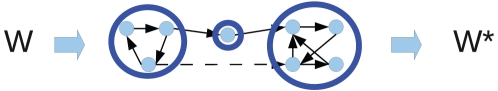
Down-ranking of unnecessary feed-forward edges. The thick rings highlight the strongly connected components of 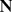. The dashed edge is *removed* from the network.

The second step of our algorithm proceeds in the following way (Figure 2):

1. Use a threshold value 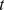 for the edge confidence (selected after several test simulations, as explained in the Results section) to extract a directed network 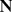 from one of the above mentioned matrices 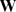.
2. Calculate the condensation of network 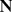, i.e. the acyclic network formed by contracting each strongly connected component of 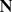 into a single vertex.
3. Obtain the subnetwork 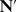 from 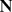 by deleting any edge such that:
  - its endpoints belong to two different strongly connected components 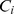 and 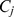, and
  - there is a path of length at least 2 between 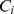 and 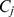 in the condensation of 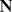.
4. For all the remaining edges in network 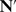, increase their corresponding weight by 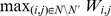, in order to ensure them a ranking higher than all the unessential edges, i.e. the edges in 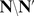.

### *In silico* data simulation

To be able to thoroughly evaluate and fine-tune the parameters of our algorithm we generated *in silico* data using our simulator developed in MATLAB.

In our model, the following nonlinear ordinary differential equation describes the evolution of the gene expression 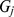:
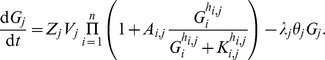


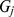
 is the gene activity (gene expression level, mRNA concentration) of gene 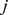, 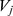 is its basal transcription rate, while 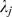 is its degradation rate constant. 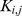 is the interaction strength of 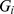 on 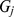, 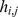 is the Hill cooperativity coefficient, and 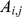 is an element of the matrix 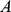 encoding the signed network structure (a positive sign corresponds to an activating regulation, while a negative one to an inhibition). Finally, 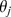 represents the biological variance (sampled from a normal distribution with 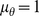 and standard deviation 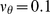), while 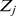 is responsible for eventually knocking-out gene 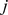. In our simulations, random networks were generated by the Erdős-Rényi (ER) algorithm [29], with various average degrees. Edge directions and signs were assigned randomly with uniform probability. Parameters 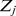, 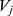, 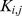, 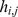, 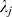 and 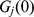 were all set equal to 1. We then calculated the *wild-type* steady state. To simulate the single-gene knockout experiments we initialize 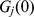 = 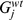 and set 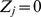 in the 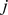-th perturbed experiment in order to simulate the knockout of gene 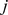; obviously 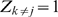 since we only simulated single-gene knockout experiments. These simulations resulted in data sets similar to the ones provided in the DREAM 4 challenges.

### Evaluation

Method effectiveness was evaluated through the calculations of the Area Under the Receiver Operating Characteristic Curve (AUC(ROC)) and the Area Under the Precision versus Recall Curve (AUC(PvsR)) [20], [24], in the same way as was done by the DREAM organizers to evaluate the submitted networks.

## Results

### Practice on the DREAM3 benchmarks

In order to make informed decisions on the choice of the weight matrices to use and to fine-tune the threshold value for the second step of our algorithm, we practiced first on the DREAM3 benchmarks [25]. The DREAM3 *In Silico* Network challenge in 2008 was very similar to the DREAM4 one. Here too GNs of different sizes (10, 50, and 100 genes) had to be inferred using steady states from wild-type, knockdown and knockout perturbations, and time series data. The kinetic equations were also similar, though in DREAM3 a deterministic model was used while in DREAM4 a stochastic one.

In order to choose which, amongst the confidence matrices 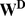, 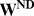, 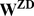 and 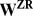, gives the most reliable initial network prediction, tests were performed on the DREAM3 benchmarks.

We initially considered both the knockout and knockdown data, but since our algorithm consistently gave better results on the knockouts (data not shown), we will here further consider only the knockout steady states.

By applying the aforementioned inferring techniques on these data, the matrices 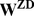 and 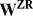 yielded the best results for the 50- and 100-gene networks, respectively for the AUC(PvsR) and for the AUC(ROC). On the other hand, a simple ordering of the edges based on the deviation from the wild type (i.e. matrix 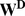) gave the best results for the small 10-gene networks for both the evaluation measures AUC(ROC) and AUC(PvsR). The results are shown in Table 2.

**Table 2:** Performances of the four considered confidence matrices on the DREAM3 networks.

	N. genes	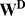	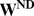	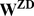	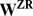
AUC(ROC)	10	**0.8194**	0.7741	0.7837	0.7901
	50	0.8444	0.8389	0.8769	**0.8875**
	100	0.8515	0.8454	0.8736	**0.8799**
AUC(PvsR)	10	**0.7028**	0.5619	0.5991	0.6732
	50	0.5396	0.4579	**0.6224**	0.6160
	100	0.5637	0.4616	**0.6200**	0.6143

Average AUC(ROC) and AUC(PvsR) for the five networks of three different sizes from the DREAM3 *In Silico* benchmarks, calculated through the confidence matrices 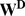, 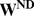, 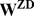 and 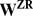. The best value of each row is bolded.

Then, given the confidence matrix 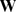, the down-ranking algorithm produces the modified matrix 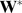 as described in the Methods section. The result of this down-ranking step depends on the chosen value for the threshold 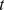. Therefore, we performed test runs at different values of 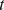 to establish the value for which the best AUCs were obtained (Table 3). We here report only the results on the larger networks as the down-ranking step had almost no effect on the reliability of the small networks. This indicates that our down-ranking approach is beneficial only for larger networks.

**Table 3:** Effect of the down-ranking algorithm on larger DREAM3 networks.

	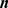	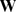	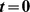	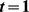	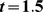	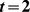	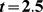	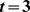	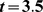	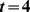
AUC(ROC)	50	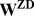	0.8769	0.8769	0.8766	0.8767	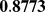	0.8773	0.8772	0.8770
		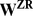	0.8875	0.8853	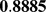	0.8884	0.8881	0.8878	0.8877	0.8875
	100	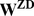	0.8736	0.8736	0.8735	0.8733	0.8735	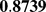	0.8738	0.8737
		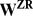	0.8799	0.8799	0.8793	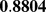	0.8804	0.8802	0.8801	0.8800
AUC(PvsR)	50	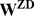	0.6224	0.6224	0.6176	0.6175	0.6411	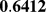	0.6377	0.6303
		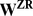	0.6160	0.5835	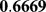	0.6666	0.6555	0.6461	63.5244	0.6259
	100	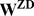	0.6200	0.6200	0.6181	61.1130	0.6222	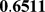	0.6456	0.6387
		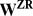	0.6143	0.6143	0.6039	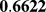	0.6603	0.6502	0.6410	0.6326

Average AUCs for the 50- and 100-gene networks from the DREAM3 *In Silico* challenge after the application of the down-ranking algorithm on matrices 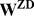 and 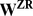 with 8 different thresholds 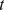. Setting 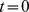 corresponds to not applying the down-ranking. The best value of each row is bolded.

Negligible differences in the AUC(ROC), but more substantial improvements in the AUC(PvsR) measures were obtained for 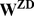 and 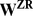, with the latter slightly exceeding the former performances. In particular, the AUCs peak for 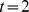 while down-ranking 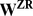, and for 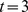 in 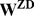 (100-gene networks). These tests suggested that using either matrix 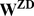 or 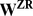 in combination with 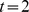 are the best choice.

### Practice on additional *in silico* data

**Figure 3:**
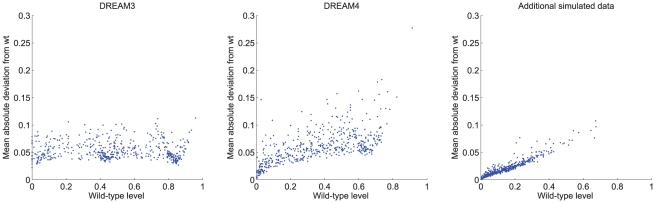
Distribution of the mean absolute deviation for three knockout datasets. Each point is the mean absolute deviation of the expression of a gene 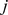 with respect to its wild type 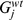, calculated as 
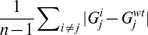
, obviously excluding the knockout of gene 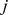 from the averaged values. Our *in silico* knockout data (right) qualitatively resembles the distribution of the five DREAM4 *InSilico_Size100* knockout datasets (middle), in contrast to those from the five DREAM3 *InSilicoSize100* knockout datasets (left).

While the DREAM3 benchmarks were of great value, there were some notable differences between the DREAM3 and DREAM4 networks and data. All the networks in DREAM3 were acyclic, while the networks considered in DREAM4 do contain cycles. Furthermore, the variance in the DREAM3 knockout data drastically differed from those in the DREAM4 knockout data. In the previous edition the mean deviation in each gene was uniform, while in the DREAM4 data it seemed proportional to the gene activity wild-type level (Figure 3). The same pattern can be observed in our self generated *in silico* data (Figure 3).

So, we decided to verify the previous choices for the confidence matrix and the threshold value on a much larger number of datasets than the DREAM3 benchmark (thus preventing overtraining), and on data which should be more similar to the DREAM4 ones.

By using our simulator, we generated 1000 100-gene networks with ER topology and average degree 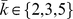 (DREAM3 100-gene networks had 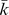 ranging from 1.2 to 5.5), and corresponding knockout datasets. The AUCs for the various confidence matrices are shown in Table 4, emphasizing that the z-score applied on the raw data (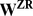) clearly appears to be the most effective method to obtain a first prediction of the network from knockout data. This choice is also supported by the test on the DREAM3 benchmarks.

**Table 4:** Performance of the four confidence matrices on additional *in silico* data.

	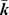	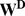	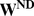	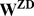	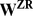
AUC(ROC)	2	0.8763	0.8829	0.9276	**0.9328**
	3	0.8223	0.8449	0.8910	**0.8972**
	5	0.7325	0.7751	0.8155	**0.8209**
AUC(PvsR)	2	0.3055	0.3839	0.5909	**0.6041**
	3	0.2602	0.3809	0.5383	**0.5519**
	5	0.2119	0.3513	0.4500	**0.4588**

Average AUCs for 1000 100-gene ER networks with average degree 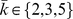 calculated through the confidence matrices 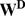, 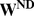, 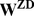 and 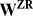. The best value of each row is bolded.

In a similar fashion, we applied the down-ranking algorithm on matrix 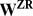, showing that a small improvement on the AUCs (especially with the PvsR one) can be obtained with threshold 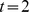 (Table 5), again in concordance with what we observed for the DREAM3 benchmarks.

**Table 5:** Effect of the down-ranking algorithm on additional *in silico* data.

	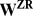 ( 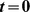 )	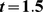	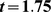	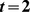	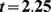	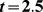
AUC(ROC)	0.8317	0.8315	0.8317	**0.8317**	0.8317	0.8317
AUC(PvsR)	0.5913	0.5793	0.5892	**0.5962**	0.5954	0.5948

Average AUCs for 1000 100-node ER networks, generated with average degree 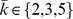, after the application of the down-ranking algorithm on matrix 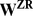 with 6 different thresholds 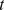. The best value of each row is bolded.

### DREAM4

**Figure 4:**
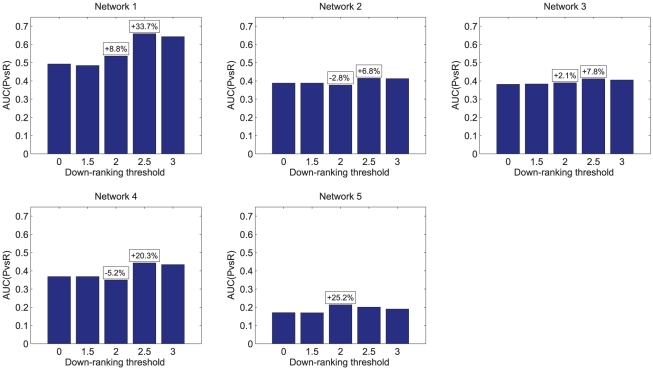
Effect of the down-ranking algorithm on DREAM4 networks. In each of the five plots, the bars show the values of the AUC(PvsR) for one of 100-gene networks from DREAM4 after the application of the down-ranking algorithm on matrix 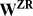 with 5 different threshold 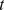. In the small boxes the most significant percentage differences with respect to the threshold 0 are shown.

After the extensive tests described above, we decided to base our predictions for the DREAM4 *In Silico* Network challenge on the z-score on raw data confidence matrix (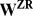), post-processed with the down-ranking algorithm using threshold 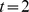. Our submission as Team ALF was the best performer at the sub-challenge 2 (100-gene networks), ranking first among 19 participants. Interestingly, now that the gold standard networks have been published, we discovered that our choice for the confidence matrix was in fact good (see Table 6), but even better predictions would have been obtained by selecting 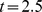 as the threshold for the down-ranking algorithm. Nevertheless, the improvement in the AUC(PvsR) obtained with the selected 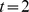 has been considerable for networks 1 and 5, as shown in Figure 4 and in Table 7, compared to those from 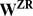.

**Table 6:** Performances of the four confidence matrices on the DREAM4 networks.

	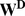	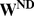	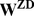	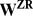
AUC(ROC)	0.7844	0.7927	0.8275	**0.8297**
AUC(PvsR)	0.2610	0.2786	**0.3710**	0.3602

Average AUC(ROC) and AUC(PvsR) for the five 100-gene networks from the DREAM4 *In Silico* benchmarks, calculated through the confidence matrices 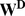, 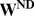, 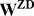 and 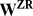. The best value of each row is bolded.

**Table 7:** Effect of the down-ranking algorithm on DREAM4 100-gene networks.

	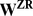 ( 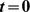 )	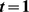	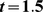	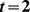	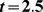	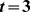	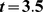	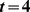
Network 1	0.4928	0.4928	0.4847	0.5361	**0.6590**	0.6428	0.6225	0.5715
Network 2	0.3880	0.3880	0.3880	0.3771	**0.4144**	0.4125	0.4052	0.3886
Network 3	0.3816	0.3816	0.3834	0.3898	**0.4115**	0.4048	0.3939	0.3895
Network 4	0.3684	0.3684	0.3684	0.3494	**0.4433**	0.4338	0.4144	0.3841
Network 5	0.1703	0.1703	0.1697	**0.2133**	0.2008	0.1902	0.1782	0.1845

Average AUC(PvsR) values for the 100-gene networks from DREAM4 *In Silico* challenge after the application of the down-ranking algorithm on matrix 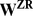 with 8 different thresholds 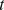. The best value of each row is bolded.

It should also be noticed that the the average node degrees in the DREAM4 networks are smaller (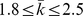) than those in DREAM3 and our simulated networks: a better estimation of the optimal threshold might have been obtained if our test networks had an average degree in the same range of the DREAM4 networks. Furthermore, we simulated data with networks generated with the ER algorithm, which have significantly different topology than those used in DREAM4. Also, note that the performances on the DREAM4 benchmarks are much more sensitive to the value of 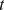 then we observed in the tests of our *in silico* data. Obviously this is due to the fact that we used a large ensemble (1000 networks) over which the performances were averaged, but it also indicates that the DREAM4 benchmarks consist of a set of networks with widely varying topologies.

## Discussion

We described an algorithm to infer gene regulatory networks from expression data, that proved to be effective by best performing at the DREAM4 *In Silico* Network challenge in the 100-gene networks sub-challenge. The proposed technique combines the advantages of the standard score in highlighting the deviation from the mean after a gene knockout, with the down-ranking algorithm that reduces the confidence initially predicted to unnecessary feed-forward edges.

Our algorithm is substantially different from the techniques used by the best performer teams of previous DREAM *In Silico* Network challenges. In particular, for DREAM2 the winning approach was fitting ordinary differential equation (ODE) models [27], [30]; for DREAM3, instead, the best method was based mainly on finding significant deviations from wild type in knockout data (so using the same primary source of information of our algorithm), but also applied ODE models on the time series for additional predictions [31].

To see how methods based on ODEs would perform on the DREAM4 data, we analyzed them with one of the best performer algorithm [27] for the DREAM2 *In Silico* Network challenges. The predictions of this algorithm on the DREAM4 100-gene networks was very poor (average AUC(ROC) = 0.5722, AUC(PvsR) = 0.0313). Note that in DREAM2 there was no noise added to the *in silico* data, while for DREAM4 both biological and experimental noise were present. Since the internal noise is propagated through gene relationships, its effect on large networks make sophisticated models (like ODEs) much less reliable than our method based on simple cause-effect logic and graph inspection.

Further increases of the performance of our algorithm may be obtained by studying the possible relationships between the selected threshold 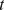 and other parameters, like the network average degree and size, the noise on the knockout data, and so on. Moreover, while the second step of our algorithm improves the inference of the so-called cascade motif [32], it should be possible to reduce also the systematic errors in the prediction of other network motifs (e.g. the fan-in and fan-out errors [32]). Finally, also the rest of the available data from the DREAM challenges (knockdowns, time series, multifactorial) may be used to refine the network prediction, but the gain would probably be small, as already shown by the DREAM3 best performer [31].

It has become unambiguously clear that systematic perturbations (e.g. experimental gene knockouts) are needed to establish the directed structure of GNs. However, systematic single-gene knockouts imply experimental requirements which are unrealistic and these experiments infeasible (and unethical) for many organisms. It is unlikely that data such as considered here will become available from real experiments. Fortunately, “systems genetics” experiments may provide an alternative. In systems genetics experiments a population under study is genotyped and gene expression profiled are simultaneously collected (possibly even including metabolomics and proteomics data [33]). It has been demonstrated that causal links in GNs can be elucidated based on these data (see [34], [35] for reviews). Genetic polymorphisms, naturally present in the populations, act as genetic perturbations: if the gene activity of a gene B is affected by a polymorphism inside another gene A, this is highly indicative for a causal effect A
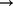
B. In fact Liu *et al.* [34] proposed a very similar strategy as the one outlined in this paper: first creating a causal influence network (but based on systems genetics data instead of knockout data like is done here) and subsequent sparsification of this network to retain only the edges corresponding to direct causal influences. In that approach each edge in the initial network was statistically tested for being supported by the data, while we were here not able to do so based on the data considered here. Down-ranking edges based on our simple graphical inspection is very useful in the context of systems genetics data as it will provide the sparsest network supporting the causal influences. This then allows methods like the one of Liu *et al.* approach to statistically identify the networks best supported by the data by adding edges, rather than removing edges from the causal influence network. Heuristic model search algorithms are strongly dependent on a good initial guess in the network space: we argue that networks which result from the algorithm described in this paper will provide a better initial guess than the initial causal influence network, as GNs are known to be sparse. In this sense, the resulting networks from our approach here should not be seen as the final prediction, but rather as inputs to more sophisticated methods involving thorough statistical testing. Nevertheless, as evidenced by its winning performance over 18 other participating teams in the DREAM4, this method can be considered state-of-the-art on its own.
